# Mistakes, Too Few to Mention? Impaired Self-conscious Emotional Processing of Errors in the Behavioral Variant of Frontotemporal Dementia

**DOI:** 10.3389/fnbeh.2017.00189

**Published:** 2017-10-17

**Authors:** Carole S. Scherling, Jessica Zakrzewski, Samir Datta, Robert W. Levenson, Arthur P. Shimamura, Virginia E. Sturm, Bruce L. Miller, Howard J. Rosen

**Affiliations:** ^1^Department of Neurology, UCSF Memory and Aging Center San Francisco, University of California, San Francisco, San Francisco, CA, United States; ^2^Department of Psychology, University of California, Berkeley, Berkeley, CA, United States

**Keywords:** error-processing, autonomic, emotion, dementia, awareness

## Abstract

Anosognosia, or lack of awareness of one's deficits, is a core feature of the behavioral variant of frontotemporal dementia (bvFTD). We hypothesized that this deficit has its origins in failed emotional processing of errors. We studied autonomic and facial emotional reactivity to errors in patients with bvFTD (*n* = 17), Alzheimer's disease (AD, *n* = 20), and healthy controls (HC, *n* = 35) during performance of a timed two-alternative-choice button press task. Performance-related behavioral responses to errors were quantified using rates of error correction and post-error slowing of reaction times. Facial emotional responses were measured by monitoring facial reactivity via video and subsequently coding the type, duration and intensity of all emotional reactions. Skin conductance response (SCR) was measured via noninvasive sensors. SCR and total score for each facial emotion expression were quantified for each trial. Facial emotions were grouped into self-conscious (amusement, embarrassment) and negative (fear, sadness, anger, disgust, contempt) emotions. HCs corrected 99.4% of their errors. BvFTD patients corrected 94% (not statistically different compared with HC) and AD corrected 74.8% of their errors (*p* < 0.05 compared with HC and bvFTD). All groups showed similar post-error slowing. Errors in HCs were associated with greater facial reactivity and SCRs compared with non-error trials, including both negative and self-conscious emotions. BvFTD patients failed to produce self-conscious emotions or an increase in SCR for errors, although they did produce negative emotional responses to a similar degree as HCs. AD showed no deficit in facial reactivity to errors. Although, SCR was generally reduced in AD during error trials, they showed a preserved increase in SCR for errors relative to correct trials. These results demonstrate a specific deficit in emotional responses to errors in bvFTD, encompassing both physiological response and a specific deficit in self-conscious emotions, despite intact awareness and correction of errors. The findings provide a potential mechanism for anosognosia and possibly other behavioral abnormalities in bvFTD and highlight the importance of studying multiple channels of reactivity to errors, including performance related responses and emotional responses, in order to understand how impaired error processing could influence behavior.

## Introduction

The behavioral variant of frontotemporal dementia (bvFTD) is a devastating neurodegenerative disorder that causes progressive deterioration in specific portions of the frontal and temporal lobes, including the orbitofrontal, anterior cingulate, and insular regions, the anterior temporal lobes and the amygdala (Perry and Miller, 2013; Rohrer and Rosen, 2013). One core feature in bvFTD is anosognosia, or lack of awareness of deficits (Rosen, 2011). Compared with other neurodegenerative disorders such as Alzheimer's disease (AD), bvFTD patients are less likely to endorse decline in their level of function, and they are significantly less accurate in rating their performance on cognitive testing (Williamson et al., 2009; Rosen et al., 2014). The lack of insight in bvFTD is so ubiquitous that this symptom was included as a required feature in the diagnostic criteria for bvFTD published in 1998 (Neary et al., 1998).

The level of anosognosia in bvFTD is particularly striking when considering their pattern of cognitive impairment. In AD, one might expect some degree of agosognosia due to profound memory impairment that could prevent patients from remembering events when their deficits have caused them problems. In contrast, bvFTD is typically associated with relatively spared episodic memory (Rascovsky et al., 2011), but is characterized by dysfunction in the brain systems that mediate socioemotional behavior, resulting in disinhibition, apathy, loss of empathy, and obsessive-compulsive behaviors (Rascovsky et al., 2011; Perry and Miller, 2013). Beyond bvFTD, anosognosia is found in a variety of neurological disorders, including other neurodegenerative diseases, and has been linked with impaired executive function and structural and metabolic changes in the frontal lobes, including the orbitofrontal cortex (Salmon et al., 2006; Rosen et al., 2010, 2014). Furthermore, recent anatomical analyses indicate that anosognosia in FTD is specifically associated with the degree of atrophy in the orbitofrontal cortex (Rosen et al., 2010; Hornberger et al., 2014). Taken together, these observations suggest that failure of the frontal systems responsible for socioemotional processing may be the chief cause of anosognosia in bvFTD.

Based on these considerations, we have theorized that failures in emotional processing contribute to anosognosia in bvFTD (Rosen, 2011). Specifically, we have proposed that errors occurring in everyday life represent opportunities for patients to reassess themselves and that altered emotional processing after an error (e.g., lack of frustration or embarrassment) may prevent bvFTD patients from attributing proper significance to their errors. The goal of the current study was to test this hypothesis by examining error processing in a laboratory setting where responses to errors could be examined directly. Prior research has extensively characterized the behavioral responses to errors and demonstrated that errors are associated with performance-related behavioral responses including slowing of reaction time on post-error trials, and error correction if subjects are instructed to do so (Rabbitt, 1966; Taylor et al., 2007). Emotional responses to errors have been examined less often, but activation of the autonomic nervous system (Hajcak et al., 2003; Wessel et al., 2011), emotional facial reactions (Mograbi et al., 2012), and activation of brain regions associated with emotional processing (Taylor et al., 2007; Wessel et al., 2011; Koban and Pourtois, 2014) have all been documented in response to errors, and some studies have indicated that emotional responding influences performance-related behavior (Taylor et al., 2007; Koban and Pourtois, 2014). Several studies have examined the effects of brain injury on error-related responses. Groups with bvFTD (O'Keeffe et al., 2007), focal frontal lobe lesions (Hoerold et al., 2013), and traumatic brain injury (O'Keeffe et al., 2004) have demonstrated reduced awareness of errors as measured by verbal reporting of mistakes, along with decreased SCRs to errors (O'Keeffe et al., 2004; Hoerold et al., 2013), but these studies did not examine post-error slowing, error correction, or emotional responses. Two studies of patients with anterior cingulate lesions found no deficit in post-error slowing (Fellows and Farah, 2005; Maier et al., 2015) or error correction (Maier et al., 2015), although a group of patients with dorsolateral frontal injury showed some deficits in both of these measures (Wessel et al., 2014). A study in AD demonstrated intact facial emotional reactivity when patients perceived their performance to be poor (Mograbi et al., 2012). Our core hypothesis was that emotional reactivity to errors as measured by physiological responses and facial reactivity would be impaired in bvFTD. Based on the prior studies, we also predicted that error correction and post-error slowing, which has been correlated with SCR (Hajcak et al., 2003; Wessel et al., 2011), could also be impaired. The study also included a group with AD. While AD is also associated with a significant degree of anosognosia, we hypothesized that the anosognosia in AD is not due to failures in emotional processing, and thus we expected AD to show normal emotional reactivity to errors.

## Materials and methods

### Participants and clinical assessment

Seventy-three participants, including 17 with bvFTD, 20 with AD, and 35 cognitively healthy normal comparison subjects (HC) were recruited from ongoing studies of bvFTD and AD (AG019724, AG032306, AG023501) at the UCSF Memory and Aging Center. Patients were referred by outside physicians, and sometimes self-referred, and all underwent neurological, neuropsychological and nursing assessment, including informant interview, and were diagnosed at a multidisciplinary consensus conference using published criteria (McKhann et al., 1984) including the Neary criteria (Neary et al., 1998) or the more recently published consensus criteria (Rascovsky et al., 2011) for bvFTD depending on year of enrollment. HCs were recruited through advertisements and community events and underwent the same diagnostic assessment as patients, and were required to have no significant cognitive complaint, no significant problems identified by a knowledgeable informant, and no significant impairments on cognitive testing. The neuropsychological battery included the Mini-Mental State Examination (MMSE; Folstein et al., 1975), a copy of the Benson complex figure (Kramer et al., 2003) to assess visuospatial function, forward and backward digit span (Wechsler, 1997), a modified Trail-making task (Kramer et al., 2003), the Stroop inhibition task (Stroop, 1935), a design fluency task (Delis et al., 2001), a 15-item Boston naming task (BNT; Kaplan et al., 1983), phonemic fluency (words beginning with the letter “D”; Kramer et al., 2003), category fluency (animals; Delis et al., 2001), the California Verbal Learning Test-Short Form (CVLT; Delis et al., 2000), and a test of memory for the Benson figure. The Clinical Dementia Rating scale (CDR) was used to quantify functional state (Morris, 1993), and the general severity of illness was represented by the CDR sum-of-the-boxes score (Daly et al., 2000). The Neuropsychiatric Inventory (NPI; Cummings et al., 1994) was used to quantify behavioral abnormalities, and the Geriatric Depression Scale was used to measure depressive symptomatology (GDS; Yesavage et al., 1983).

This study was carried out in accordance with the recommendations of the UCSF Committee on Human Research with written informed consent from all subjects. All subjects gave written informed consent in accordance with the Declaration of Helsinki. The protocol was approved by the UCSF Committee on Human Research.

### Apparatus and setup

Testing took place in a in a ten-by-ten foot room, and the experiment was controlled from an adjacent room. Stimuli were presented on a computer monitor (Dell model # U2212HMC, 21 ½″ diagonal), attached to a stimulus presentation computer (Dell Optiplex 980 with Windows 7 32-bit operating system), running E-Prime Professional 2.0 (www.pstnet.com) for experimental control. Auditory stimuli were presented to the participant using Sony MDR 7506 professional large diaphragm headphones with a frequency response of 10 Hz–20 kHz (www.sony.com). Button presses were recorded using a two-button response box.

The testing room was equipped with a Panasonic remote pan/tilt/zoom camera (resolution 704 × 490 with 29.97 frames per second), installed in a bookshelf directly across from, and clearly visible to the participant and controlled remotely using a Picolo U4 H.2634 video board. Video signal was passed to a data capture computer (Dell Precision T1500 with Windows 7 64-bit operating system) with Noldus Media Recorder 2 software (www.noldus.com) and a Panasonic WJ-MP-204C data multiplex with WV-CU360 system controller. Video was captured using the Noldus Observer XT software package. Audio for subject vocal responses was collected using a Shure push-to-talk microphone (www.shure.com) and was integrated with video through a RU-MX5ML mixer (www.rdlnet.com).

SCR was measured in microSiemens (μS) on a continuous basis by attaching two 1,081 FG-DIN Ag/AgCl sensors prepared with Biogel electrode gel (UFI inc., Morro Bay, CA) to the ventral surface of the middle phalanges on the middle and index fingers of the participant's non-dominant hand. The sensors were connected to an SAI bioamplifier (0.5 V constant voltage with a sensitivity of 600 pS, SAI Inc., Hauppauge, NY), which was in turn connected to a Biopac UMI100 to be digitized. The signal was then fed to the data capture computer for subsequent processing with Acknowledge data acquisition software (version 4.2, www.biopac.com).

Finally, timing of stimulus onset and participant button-presses were passed from the stimulus computer to the data capture computer and integrated with physiological signal in Acknowledge and video signal in Observer XT.

After giving informed consent, participants were seated 4.25 feet in front of the stimulus presentation monitor, the button box was placed in front of their dominant hand and the SCR sensors were attached to their non-dominant hand. Headphones were placed over their ears, and their hearing was tested using a series of 1 and 2 khz tones. Participants were asked to indicate the onset and offset of the tones by raising and lowering their hands. Those failing the hearing test were excluded from the study.

### Experimental procedures

#### Startle

Prior to beginning the experimental task, participants were exposed to a stimulus designed to elicit a startle response to provide a measure of basic physiological responding. Participants were asked to sit quietly for 2 min. After 34 s, a 105 dB white noise burst was played for 500 ms without warning.

#### Experimental task

Participants completed a modified version of the stop-signal task that is referred to as the stop-change task (Boecker et al., 2013). While the stop-signal and stop-change task are often used to study action planning and execution, our goal was not primarily to assess task performance, but rather to choose a task that would maximize our ability to study responses to errors. Thus, the requirements for the task were: (1) to reliably elicit errors in controls and patients, (2) to permit manipulation of parameters in order to produce roughly equal rates of errors across groups, and (3) to permit an inter-trial interval long enough to measure SCR (at least 3 s). The motivation to equalize error rates was that widely disparate rates of errors across groups might confound interpretation of differences in error-related reactivity. After extensive piloting of various tasks, the stop-change task was identified as the best for these purposes.

Each trial began with a white “X” (Courier New font, 120 pt size) displayed for 500 ms on a black background. The participant was instructed that when the “X” appears, they should press the button labeled “1” on the button box as quickly as possible. This constitutes a complete trial for the 80% of the trials (X-trials), and the subjects viewed a blank screen until the next trial (duration of 6 s). On 33% of the trials, the “X” was followed by a beep tone (Beep-trial, 1,000 Hz, duration of 500 ms). The participant was instructed that on these types of trials, they should not press button 1, but should instead press the button labeled “2.” Thus, the encouraged strategy is to wait just long enough that the beep tone should have sounded before pressing button number 1. They are also instructed that if they press button 1 and then hear the beep tone, indicating that they have made a mistake, they should press button 2 immediately to correct their mistake. The tone was initially set to follow the offset of the “X” by 500 ms. Subsequent delays varied with performance, such that after a correct response (i.e., withholding the response until the beep tone has sounded), the duration was lengthened by 50 ms, and after an incorrect response the delay was shortened by 50 ms with the minimum delay being 300 ms and the maximum being 2,500 ms. This design was chosen to allow the proportion of errors to be roughly the same across diagnostic groups. The task began with a practice block consisting of 6 trials, followed by two experimental blocks of 60 trials each. Twenty Beep-trials in each block were randomly dispersed among the trials, with the restriction that Beep-trials would have to be separated by at least one X-trial. Participants who could not learn the task (e.g., never waited for the tone, always waited to respond until after the tone sounded, failed to correct their mistakes…) were not included in the analysis, nor were any participants who corrected fewer than 50% of their errors during the task.

#### Self-assessment task

After hearing a description of the experimental task, each subject was asked to predict the number of trials on which they would make errors. At the end of the task, they were not told how they had performed but were asked to estimate the number of trials on which they had actually made errors.

### Measures

#### Button-press responses

All button presses were captured with E-prime, allowing the calculation of the proportion of correct and incorrect trials, as well as the proportion of incorrect trials that were corrected. Reaction time (RT) was calculated as the number of milliseconds between the presentation of the X and the first button press. To assess changes in RT after errors, we created change scores by subtracting the RT on every Beep-trial from the RT on the next trial. This calculation allows comparison of RT change scores following errors with RT change scores following correctly performed Beep-trials.

#### Facial emotional behavior

Facial behavior was coded using the Emotional Expressive Behavior System (EEB; Gross and Levenson, 1993). Coders were not involved in the experimental session and were blind to diagnosis. Eight emotions were coded on an intensity scale of 0–3 (0 = no emotion, 1 = mild, 2 = moderate, 3 = severe): anger, contempt, disgust, fear, happiness/amusement, embarrassment, sadness, and surprise. Coders reviewed the video for the entire experimental task and coded the intensity of each emotion on a second-by-second basis, allowing the calculation of duration. Once all coding was complete we calculated an emotion intensity-by-duration product score (IxDscore) for each trial. To reduce the number of statistical tests, we created facial reactivity summary scores representing the degree of negative emotions (sum of IxDscores for anger, contempt, disgust, fear) and self-conscious emotions (sum of IxDscores for embarrassment and happiness/amusement) for each trial. These groupings were chosen based on prior studies indicating that bvFTD is associated with impaired self-conscious emotions using similar categorization (Sturm et al., 2006).

#### Skin conductance

We calculated the maximum SCR for all trials. For X-trials, the SCR was assessed beginning at the onset of the X, and for Beep-trials, the SCR was assessed beginning at the onset of the beep tone. For the startle response, the SCR was assessed beginning at the startle and extending for 5 s after. The magnitude of the startle response was calculated as the peak SCR minus the SCR for the 1 s preceding the startle onset. The raw SCR data showed a large positive skew, so the SCR data were log-transformed to better approximate a normal distribution, which is a common approach for analyzing SCR data (Boucsein, 2012).

### Statistical analysis

Variables consisting of a single observation per subject (such as demographic variables, the number of errors and proportion of errors corrected) were compared using Chi-square analyses and ANOVA as appropriate. Basic physiological reactivity was examined by comparing SCR for the startle across groups using ANOVA. Trial-by-trial data, which represent repeated measures within subjects, were analyzed using linear mixed effects (LME) models. Trials were separated into three types: X-trials, correct Beep-trials, and incorrect Beep-trials. Our hypothesis was that bvFTD would have a deficit in emotional responding to errors. This was examined using an LME model that included diagnosis and trial type as fixed effects. For facial reactivity, we used IxDscore as the outcome variable and included emotion type as a fixed variable and the interaction between emotion type and diagnosis. The model included random intercepts, nesting by emotion type. Age and sex were included as covariates. Similar analyses were performed using SCR and post-error slowing (using post-correct Beep-trial RT as an additional covariate) as outcome variables. All statistical analyses were performed using R (R Core Team, 2016). LME analyses were performed using the nlme package (Pinheiro et al., 2016). *Post-hoc* tests were performed as pairwise differences with Tukey adjustments using the lsmeans package (Lenth, 2016).

## Results

### Demographics and clinical characteristics

Basic demographics, neuropsychological and behavioral scores are summarized in Table 1. There were no statistically significant differences in age or sex distribution across groups. Education levels were significantly different across groups, with the HC group having more years of education compared to the AD group. Both patient groups were significantly worse on multiple cognitive tests compared with HC, including the MMSE, Trails task, digit spans forward and backward, all three fluency tasks, and picture naming. Only the AD group was significantly worse on calculations and picture recall compared with HC, and the AD group had a slightly higher GDS score compared to HC. The bvFTD group scored significantly higher on the CDR-SB and NPI compared with the AD group, but there were no additional differences in cognitive performance between bvFTD and AD.

**Table 1:** Demographic, functional, cognitive and behavioral characteristics of study groups.

	**HC**	**AD**	**bvFTD**	**Statistical analysis**	**Significant contrasts**
Age (years)	67.8 (6.5)	67.9 (11.9)	62.1 (8.2)	*F*_(2, 69)_ = 2.86, NS	NA
Gender (M/F)	15/20	11/9	9/8	χ^2^ = 0.92, NS	NA
Education (yrs)	18.3 (2)	16.4 (2.3)	16.5 (4.1)	*F*_(2, 68)_ = 4.32, *p* = 0.02	AD < HC
Handedness (R/L/Ambi)	28/6/1	12/1/0	16/1/0	χ^2^ = 3.73, NS	NA
CDR-SB	0.01 (0.08)	4.1 (2.0)	6.4 (2.0)	*F*_(2, 69)_ = 128.5, *p* < 0.001	bvFTD < HC, AD < HC, bvFTD<AD
MMSE	29.3 (1.1)	23.6 (3.4)	25.5 (2.3)	*F*_(2, 54)_ = 30.8, *p* < 0.001	bvFTD < HC, AD < HC
CVLT-LD^a^	–	1.9 (2.2)	3 (3.1)	*t*_(33)_ = 1.17, NS	NA
Trails time (seconds)	23.7 (9.8)	68.6 (37.8)	72.4 (41.2)	*F*_(2, 56)_ = 18.2, *p* < 0.001	bvFTD > HC, AD > HC
Benson recall	15.6 (1)	12.9 (4.6)	14.9 (1.3)	*F*_(2, 54)_ = 4.8, *p* = 0.01	AD < HC
Calculations	4.8 (0.4)	3.6 (1.2)	4.3 (0.9)	*F*_(2, 60)_ = 11.3, *p* < 0.001	AD < HC
Design fluency	12 (3)	6.3 (3.5)	6.8 (4.1)	*F*_(2, 58)_ = 18.5, *p* < 0.001	bvFTD < HC, AD < HC
Phonemic fluency	15.8 (4.3)	10.9 (4.1)	8.3 (5.2)	*F*_(2, 55)_ = 14.1, *p* < 0.001	bvFTD < HC, AD < HC
Category fluency	23.6 (4.1)	11.9 (6)	12 (8.2)	*F*_(2, 55)_ = 24.4, *p* < 0.001	bvFTD < HC, AD < HC
Digit span forward	7.4 (1.3)	5.2 (1)	5.9 (1.1)	*F*_(2, 49)_ = 17.9, *p* < 0.001	bvFTD < HC, AD < HC
Digit span backward	5.9 (1.6)	3.6 (1.1)	4 (1)	*F*_(2, 49)_ = 15, *p* < 0.001	bvFTD < HC, AD < HC
Boston naming test	14.5 (0.9)	11.4 (2.8)	12.1 (4.2)	*F*_(2, 53)_ = 7.7, *p* = 0.001	bvFTD < HC, AD < HC
GDS	2.3 (2.5)	7.2 (5.3)	4.8 (6.5)	*F*_(2, 53)_ = 5.9, *p* = 0.004	AD > HC
NPI total	NA	24 (10.9)	46.5 (19)	*t* = 3.84, *p* < 0.001	bvFTD > AD

a *LD, long delay*.

### Experimental task performance

Task performance is summarized in Table 2. There were significant differences in error commission across diagnostic groups [*F*_(2, 69)_ = 6.54, *p* < 0.001], with bvFTD making significantly more errors compared with HC (+4.3 errors per session, 95% CI [0.7, 8], *p* = 0.003) and compared with AD (+5.9 errors per session, 95% CI [1.9, 10], *p* = 0.002). The difference in number of errors between AD and HC was not significant (*p* = 0.52). BvFTD also showed the shortest reaction times while AD showed the longest (+426 ms AD vs. bvFTD, 95% CI [142.2, 709.8], *p* = 0.01). Differences in reaction times between controls and dementia groups were not statistically significant for bvFTD (*p* = 0.36) or AD (*p* = 0.11).

**Table 2:** Performance on stop-change task across groups.

	**HC**	**AD**	**bvFTD**
Number of errors/session (*SD*)	5.5 (3.7)	3.9 (4.4)	9.8 (7.9)
Mean RT (*SD*)	1748.9 (573.0)	1996.1 (812.0)	1570.2 (709.9)
Change in RT post-correct (*SD*)	375.8 (435.6)	599.6 (439.8)	209.9 (475.2)
Change in RT post-error (*SD*)	602.0 (352.2)	1172.8 (742.4)	698.8 (714.3)
Correction Rate as percent (*SD*)	99.4 (3.5)	74.8 (32.3)	94.0 (11.2)

Reaction times were longer after errors than after correctly-performed Beep-trials (Table 2), with a significant trial-type by diagnosis interaction [*F*_(2, 2621)_ = 8.74, *p* < 0.001]. *Post-hoc* testing revealed that both patient groups showed more post-error slowing than the HC group. The HC group slowed by 301.7 m more after errors than they did after correct Beep-trials (95% CI [205.9, 397.5], *p* < 0.001), while AD slowed by 630 ms more post-error compared with post-correct trials (95% CI [475.8, 784.2], *p* < 0.001). BvFTD slowed by 556.4 ms more post-error compared with post-correct trials (95% CI [423.4, 689.5], *p* < 0.001). The degree of post-error slowing was smaller in HC compared to bvFTD (−254.7 ms, 95% CI [−415.4, −94.0], *p* = 0.002) and AD (−328.3 ms, 95% CI [−506.2, −150.4], *p* < 0.001), with no statistically significant difference between bvFTD and AD (*p* = 0.47).

All groups corrected the majority of their errors, with HC correcting the highest percentage at 99.4% (Table 2). There was a statistically significant main effect of diagnosis [*F*_(2, 62)_ = 11.55, *p* < 0.001]. BvFTD corrected 94.0% of their errors (*p* = 0.53 compared with HC) and AD corrected 74.8% of their errors (difference of −24.6% vs. HC, 95% CI [12.3, 37.0], *p* < 0.001; difference of −19.2% vs. bvFTD, 95% CI [5.0, 33.4], *p* = 0.005).

Thus the overall pattern of performance-related behavior in bvFTD was that they made more errors than other groups, but corrected the majority of their errors and slowed down appropriately after making errors. AD corrected the fewest errors but also appropriately slowed after making errors.

### Facial emotional and physiological reactivity to errors

**Figure 1:**
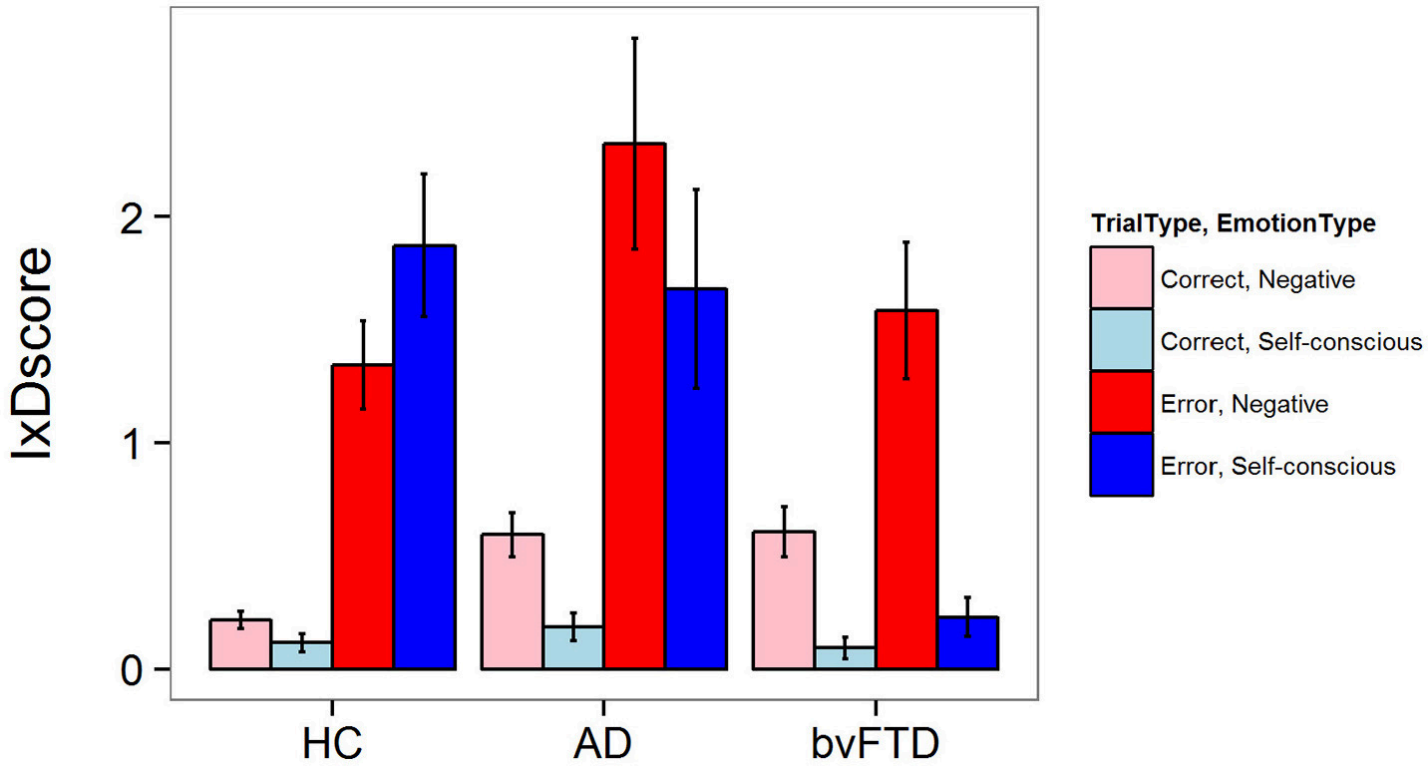
Facial emotional reactivity Beep Trials.

There was a significant three-way interaction between diagnosis, trial type, and emotion type for facial emotional reactivity [*F*_(2, 5502)_ = 7.11, *p* = 0.0008]. As illustrated in Figure 1, HCs generated both negative and self-conscious emotional responses that were larger after errors than after correct Beep-trials. This pattern of reactivity was seen in AD as well, but in bvFTD there was little generation of self-conscious emotion in response to errors. In *post-hoc* comparisons, bvFTD displayed less self-conscious emotion on error trials when compared to controls (−1.55 IxD units 95% CI [0.93, 2.16], *p* < 0.001) and AD patients (−1.61 IxD units, 95% CI [0.86, 2.36], *p* < 0.001). There were no statistically significant differences across diagnoses for negative emotions during error trials, although the increase in negative emotions in AD compared with HC approached significance (+0.8 IxD units, 95% CI [0.11, 1.49], *p* = 0.07). There were no statistically significant differences across groups for correct trials for either emotion type.

**Figure 2:**
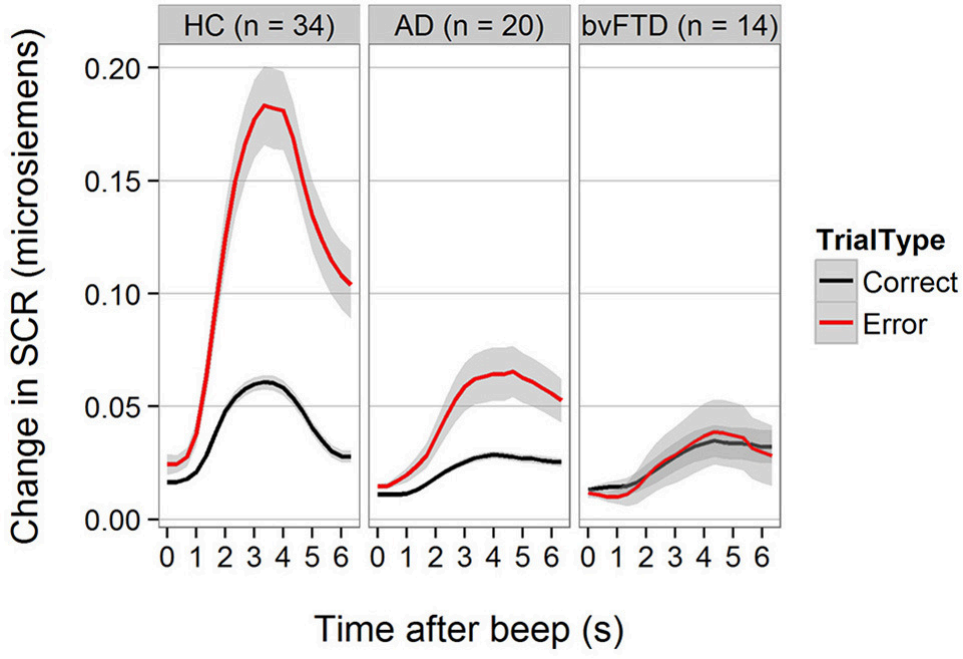
Skin conductance response for correct and error trials across groups.

For SCR, the diagnosis-by-trial-type interaction was also statistically significant [*F*_(2, 2597)_ = 18.77, *p* < 0.001]. Figure 2 demonstrates that HCs generated higher SCRs on error trials compared with correctly performed Beep-trials. The increase in SCR was smaller but still present in AD, but not detectable in bvFTD. The difference between HC and AD was statistically significant (−1.01 units, 95% CI [0.44, 1.59], *p* < 0.001) as was the difference between HC and bvFTD (−1.23 units, 95% CI [0.61, 1.87], *p* < 0.001). The difference between bvFTD and AD was not statistically significant (*p* = 0.8).

### Startle reactivity

**Figure 3:**
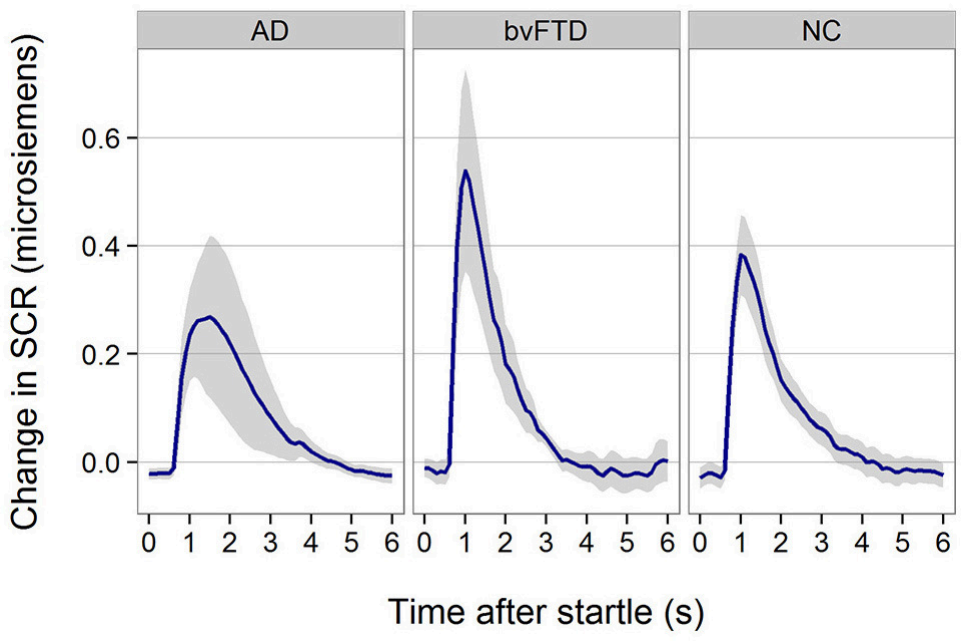
Skin conductance response for startle stimulus across groups.

All patient groups showed SCR responses to startle (Figure 3). Although, the response appeared smaller in AD, there was no statistically significant difference across groups (*p* = 0.29).

### Self-assessment

**Figure 4:**
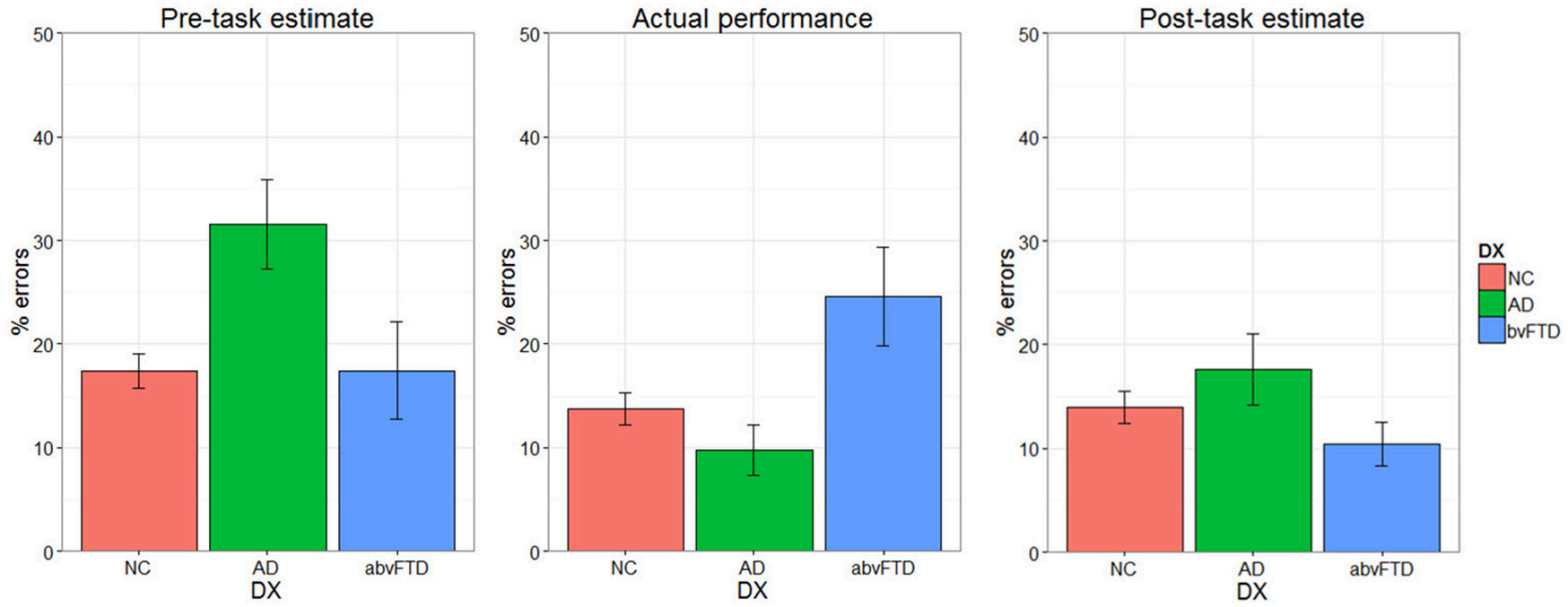
Self-assessment and performance.

**Figure 5:**
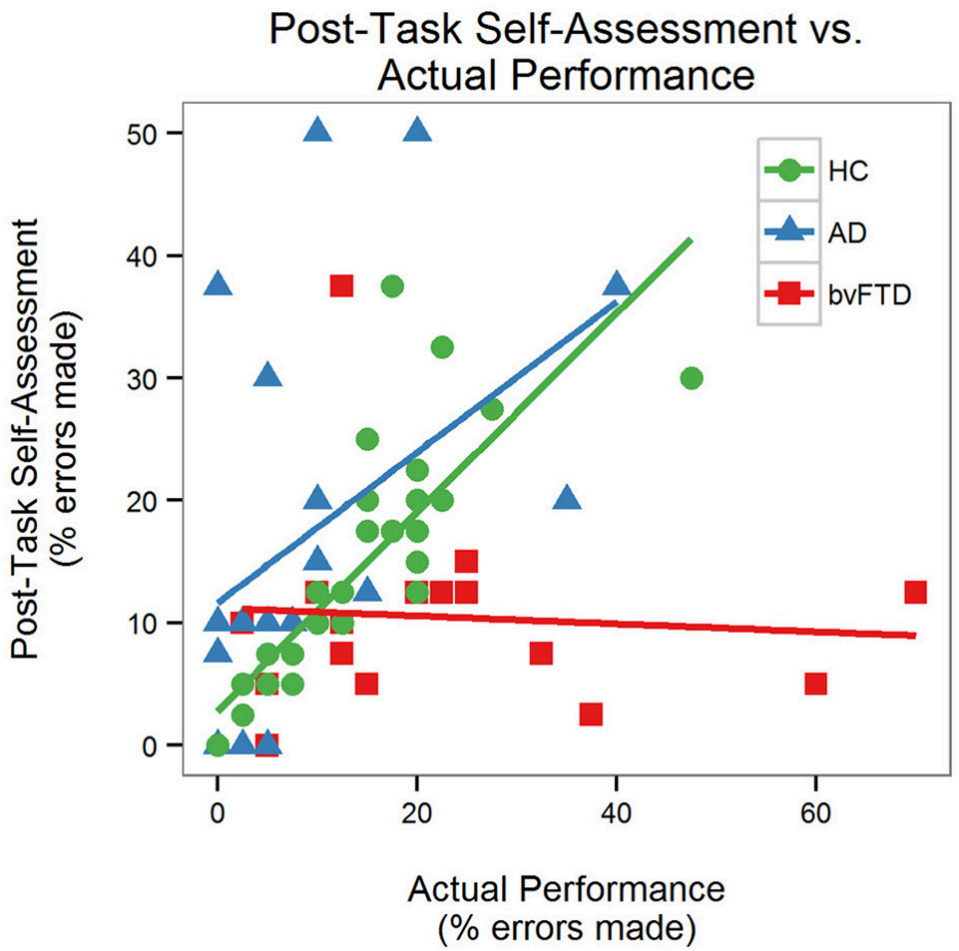
Self-assessment and performance correlation.

There was a significant performance-by-group interaction [*F*_(2, 64)_ = 6.08, *p* = 0.004] for self-assessment, indicating that the effect of actual performance on post-task self-assessment was different across diagnostic groups. Prior to the task, the AD group estimated that they would make more errors than the HC and bvFTD groups (Figure 4). The HC and AD groups made fewer errors than they predicted whereas the bvFTD group made more errors than they predicted. Both HC and AD lowered their estimates after completing the task. The bvFTD group, however, also lowered their estimate after the task despite making the largest number of errors, thus creating less agreement between their estimates and their actual performance. As illustrated in Figure 5, there was a positive correlation between performance and post-task self-assessment in HC and this relationship appeared to be somewhat present in AD but not bvFTD. *Post-hoc* testing revealed that actual performance strongly predicted post-task assessment in HC [*F*_(2, 32)_ = 32.77, *p* < 0.001], but this relationship was not significant in AD (*p* = 0.14) or bvFTD (*p* = 0.7).

### Effects facial reactivity and physiology on reaction time

We examined whether facial reactivity predicts the degree of post Beep-trial slowing. There was a statistically significant diagnosis by trial type interaction with IxDscore for self-conscious emotions [*F*_(2, 2615)_ = 17.0, *p* < 0.001] in predicting RT. Increased self-conscious facial reactivity was associated with more slowing in both HC [*F*_(1, 1353)_ = 8.17, *p* = 0.004] and bvFTD [*F*_(1, 720)_ = 6.45, *p* = 0.01]. Conversely increased self-conscious facial reactivity was associated with less post-error slowing in AD [*F*_(1, 720)_ = 12.88, *p* < 0.001]. There was no statistically significant relationship between negative emotions and slowing. For SCR, we did not identify any interactions between diagnosis, trial type, and SCR (*p* = 0.13) or trial type and SCR (*p* = 0.14) but there was a statistically significant main effect of SCR on slowing [*F*_(1, 2485)_ = 18.8, *p* < 0.001], with higher SCR predicting more slowing regardless of trial type. The effects of trial type in predicting reaction time were still highly statistically significant even in models that included SCR and self-conscious facial reactivity [*F*_(1, 2615)_ = 147.8, *p* < 0.001] and SCR [*F*_(1, 2485)_ = 132.4, *p* < 0.001], indicating that slowing on errors is mediated by other factors in addition to emotional reactivity.

### Effects of facial reactivity and physiology on self-assessment

Because actual performance strongly predicted self-assessment in HC, we included actual performance in an interaction term to see if emotional variables modified this effect. For self-conscious facial reactivity, there was a statistically significant interaction between IxDscore, performance, and diagnosis [*F*_(2, 51)_ = 6.46, *p* = 0.003]. *Post-hoc* analysis demonstrated that neither HC (*p* = 0.36) nor bvFTD (*p* = 0.32) showed a significant interaction between IxDscore and actual performance in assessing their performance, whereas in AD increased IxDscore caused patients to lower their estimates [*F*_(1, 10)_ = 20.65, *p* = 0.001]. For negative facial reactivity, there was no interaction between performance, facial reactivity and diagnosis (*p* = 0.31) or between performance and facial reactivity (*p* = 0.39) or main effect of facial reactivity (*p* = 0.53) on self-assessment. Similarly for SCR, there was no interaction between actual performance, SCR and diagnosis (*p* = 0.79) or between performance and SCR (*p* = 0.44) or main effect of SCR (*p* = 0.09) on self-assessment.

## Discussion

The goal of this study was to assess emotional reactivity to errors in bvFTD and AD. As predicted, we identified deficits in autonomic and facial emotional reactivity to errors in bvFTD. The deficit in in bvFTD was limited to particular types of emotional reactivity, in that bvFTD patients failed to generate self-conscious emotions or SCRs after errors despite being aware of them (as indicated by corrections), but they could generate normal levels of negative emotions. The impairment in emotional reactivity was also specific to a diagnosis of bvFTD, because self-conscious emotional reactivity was lower in bvFTD compared AD, who showed a level of cognitive dysfunction similar to the bvFTD group but showed no deficit in self-conscious emotion. Furthermore, whereas HC and AD patients adjusted their estimate of performance based on their actual performance, bvFTD patients failed to appropriately adjust their estimates after completion of the task. Surprisingly, bvFTD patients displayed normal immediate adjustments to performance in response to errors, including correcting nearly all of them and generating normal post-error slowing of reaction times. These findings have significant implications for the clinical presentation of bvFTD as well as the organization of self-monitoring processes in the brain.

Our findings are consistent with prior studies, which showed that bvFTD patients are impaired at generating facial responses such as amusement and embarrassment, but can generate negative emotional responses in embarrassing situations (Sturm et al., 2006, 2008). The current study extends previous work by demonstrating that this deficit applies to error monitoring, and it is not present in AD. The results suggest that when bvFTD patients commit errors, they can recognize them as errors but are impaired at attributing emotional significance to them. In daily life this could prevent patients from acknowledging the impact of their disease on their capabilities and adjusting their activities accordingly, and thus constitute a basis for anosognosia. Lack of emotional concern has been previously hypothesized as a contributor to anosognosia in bvFTD (Mendez and Shapira, 2011; Rosen, 2011). The potential impact of impaired emotional processing on self-assessment was illustrated in our study by the finding that bvFTD patients failed to normally adjust their self-assessment based on their actual performance. This is consistent with a prior study from our group, which demonstrated that bvFTD patients fail to adjust their estimate of their abilities even after overt feedback about exactly how they performed on a task (Rosen et al., 2014). The absence of an effect of self-conscious emotional reactivity on self-assessment in HC was likely due to the fact that actual performance was very accurately recalled, as indicated by its strong effect on self-assessment, but the effect of self-conscious emotion was detectable in AD, where it caused patients to lower their estimation of their performance.

The preservation of emotional reactivity in AD is consistent with prior work showing that facial reactivity is intact in AD when they are performing poorly on cognitive tasks (Mograbi et al., 2012), although that study did not characterize the specific emotional expressions or measure emotional reactions on a trial-by-trial basis. Our study provides further evidence that emotional responding to errors is intact in AD. This suggests that impaired emotional responding to errors is not likely to be an important contributor to anosognosia in AD. However, AD patients did show some impairment in error monitoring, as indicated by their decreased frequency of error correction, and their decreased ability to use their actual performance to guide their self-assessment, as compared with HC. This may represent an inability to track their errors, but the finding is open to multiple interpretations. For instance, lack of error correction may result from momentary confusion, or momentary forgetting of the task instructions. It is also possible that patients with AD felt overwhelmed by the task and decided that they should forgo error correction to focus more on other aspects of task performance. Thus, while this finding may provide clues to anosognosia in AD patients, additional studies would be required to address these different interpretations and more clearly link error performance to self-assessment in AD.

Our results also have broader implications for the role of emotion in error processing. Although, no prior studies that we are aware of have examined facial emotional reactivity to errors on a trial-by-trial basis, previous studies have demonstrated autonomic changes in association with errors, particularly if they are perceived by the subject (Hajcak et al., 2003; Wessel et al., 2011). Furthermore, fMRI (Taylor et al., 2006), and intracranial recording studies (Pourtois et al., 2010) have demonstrated activation of emotion-related brain regions such as the medial prefrontal cortex and amygdala in association with errors. We further characterized activation of specific types of emotions, demonstrating both negative and self-conscious emotional reactivity after errors. The role played by emotional activation in error monitoring has not been well-established. Some authors have hypothesized that autonomic responses represent an orienting response that contributes to awareness (Wessel et al., 2011). Our finding that SCR was associated with post-trial reaction time for beep-trials regardless of whether an error was made or not suggests that autonomic responses may indeed represent an arousal response that influences performance. However, bvFTD patients did not generate an SCR or self-conscious facial expressions on error trials yet their error correction rates did not differ significantly from controls. This result does not support the theory that the arousal associated with SCR is necessary for awareness of errors. Other models suggest that emotional activation marks the significance of errors in the context of a motivational framework, in some cases attributing errors with a significance that can lead to reassessment of one's worth (Taylor et al., 2007). This idea is supported by the fact that bvFTD patients did not adjust their self-assessment based on their performance. Because bvFTD patients showed a deficit in self-conscious emotional responding but no impairment in negative emotional responding, we propose that self-conscious emotions are particularly important for this type of self-assessment. This is also supported by the fact that self-conscious emotions, but not negative emotions, had an impact on self-assessment in AD. Self-conscious emotions are cognitively complex, requiring an evaluation of the self in relation to social expectations (Tangney, 1999). They emerge relatively late in phylogeny and ontogeny (Lewis et al., 1989) and often occur when one's behavior violates social norms (Lewis, 1995). It is hypothesized that activation of self-conscious emotion helps motivate reparative actions (Miller and Leary, 1992; Keltner and Buswell, 1997; Keltner and Anderson, 2000). Many errors, including those observed in the laboratory setting, occur in a social context, which may enhance the activation of self-conscious emotions.

If the role of self-conscious emotion in error monitoring is to motivate reparative action, what types of action might it mediate? Although, error correction and post-error slowing are well established responses to errors, the consistency with which bvFTD patients corrected their errors and the fact that they showed a normal degree of post-error slowing suggests that these performance-related adjustments are not dependent on self-conscious or autonomic activation. This is supported by the fact that the association between SCR and slowing reaction time was not specific to errors in our study and, although self-conscious emotional responding did enhance post-error slowing, the effect of errors on reaction time was strong and statistically significant even after controlling for the effect of SCR and the degree of self-conscious facial reactivity. Furthermore AD patients, who had the most difficulty tracking their errors, actually showed less slowing after trials where they had the strongest self-conscious facial reactivity, suggesting that strong emotional reactivity can impede these performance-related behaviors. The dissociation between autonomic and facial emotional reactivity and performance-related behaviors is somewhat surprising given that a large body of research has demonstrated that errors activate frontal regions, in particular anterior cingulate cortex (Taylor et al., 2007) and these activations correlated with adjustments in performance after errors. BvFTD is associated with anterior cingulate and medial prefrontal cortex atrophy (Tartaglia et al., 2011; Rohrer and Rosen, 2013) and thus they might be expected to show deficits in these behaviors. Two prior studies of patients with focal lesions in the anterior cingulate region demonstrated intact post-error slowing, which is consistent with our results (Fellows and Farah, 2005; Maier et al., 2015). Thus we propose that emotional reactivity in the context of errors might motivate higher-level adjustments such as choosing new strategies, retraining on a task, or abandoning a task altogether, rather that trial-by-trial adjustments of performance. The medial and orbital portions of the frontal lobe contain several sub-regions that participate in various aspects of error monitoring, and multiple components of error-related activity in the brain have been identified (Taylor et al., 2007; Koban and Pourtois, 2014), some of which may relate to the self-conscious emotional reactivity identified here. In fact, decreases in self-conscious emotion in response to embarrassing situations have been liked to anterior cingulate atrophy in prior studies of neurodegenerative disease (Sturm et al., 2012).

In summary, we examined multiple aspects of error responding that co-occur in normal individuals. By studying patients with neurodegenerative diseases affecting different neural systems, we were able to dissociate some of these responses in a way that provides insight into their relationships and potential roles in error processing. We found that, even though autonomic responses were higher on error trials, both post-error slowing and error correction (indicating overt awareness) can occur normally even in the absence of autonomic or self-conscious emotional reactivity. This suggests that, while autonomic reactivity may contribute to adjustment of performance normal behavior, it is not necessary to support adjustment of reaction times in response to errors or for awareness that an error has just occurred. Because impairment in self-conscious reactivity was specific to bvFTD, who also had the worst ratings of their overall performance, our findings suggest that self-conscious responding may play a critical role in higher levels of self-assessment, supporting some prior theories about the role of emotions in error processing. The reduction in SCR seen in bvFTD may also be a reflection of altered emotional reactivity to errors, but it appears to be less correlated with overall self-assessment. In the context of neurodegenerative disease, high level adjustments in behavior are critical to avoid continuing to pursue tasks that put patients and those around them at risk, such as working when one is not capable of doing a job, or driving when it is no longer safe. Self-conscious emotional processing may be a critical component supporting the ability to make these difficult adjustments.

## Author contributions

CS: Data collection, analysis, manuscript preparation. JZ, SD: Data collection, data analysis, manuscript review, and editing. RL, AS: Study design, consultation on analysis, manuscript review and editing. VS: Consultation on data collection and analysis, manuscript review and editing. BM: Secure funding, characterization of participants, review and edit the manuscript. HR: Secure funding, characterization of participants, study design, study direction, review and edit the manuscript.

### Conflict of interest statement

The authors declare that the research was conducted in the absence of any commercial or financial relationships that could be construed as a potential conflict of interest.
